# Leveraging machine learning to assess market-level food safety and zoonotic disease risks in China

**DOI:** 10.1038/s41598-022-25817-8

**Published:** 2022-12-15

**Authors:** Qihua Gao, Retsef Levi, Nicholas Renegar

**Affiliations:** Sloan School of Management, Massachusetts Institute of Technology, 100 Main Street, E62, Cambridge, MA 02142 USA

**Keywords:** Diseases, Risk factors

## Abstract

While many have advocated for widespread closure of Chinese wet and wholesale markets due to numerous zoonotic disease outbreaks (e.g., SARS) and food safety risks, this is impractical due to their central role in China’s food system. This first-of-its-kind work offers a data science enabled approach to identify market-level risks. Using a massive, self-constructed dataset of food safety tests, market-level adulteration risk scores are created through machine learning techniques. Analysis shows that provinces with more high-risk markets also have more human cases of zoonotic flu, and specific markets associated with zoonotic disease have higher risk scores. Furthermore, it is shown that high-risk markets have management deficiencies (e.g., illegal wild animal sales), potentially indicating that increased and integrated regulation targeting high-risk markets could mitigate these risks.

## Introduction

Numerous twenty-first century pandemics, such as the 2003 severe acute respiratory syndrome (SARS-CoV) outbreak and repeated avian flu outbreaks (H5N1, H7N9), have raised public attention to human health risks of *zoonotic diseases*, which transmit from animals to humans. Other outbreaks such as Middle East Respiratory Syndrome (MERS-CoV), swine influenza virus (SIV), brucellosis, have all originated from zoonosis^1–8^.

Notably, many of these outbreaks, including the 2003 SARS-CoV outbreak, were associated with large Chinese wholesale markets (WSMs) and smaller wet markets (WMs), both of which play a unique and prominent role as consolidation and distribution points in China’s agricultural supply chains^9,10^. While the origin of COVID-19 is debated, one hypothesis ties the first known cluster of COVID-19 to the *Wuhan Huanan Seafood Market*, and a later cluster to the *Beijing Xinfadi Seafood Market*^11,12^*.* Likewise, SARS-CoV was associated with markets in Foshan, brucellosis cases in 2005–2010 were linked to pork markets in Guangdong, and H7N9 cases in 2013–2017 were associated with poultry markets in Shanghai and Anhui^3,4,7,13^. Existing hypotheses suggest that illegal sale of exotic wild animals and sanitation issues at markets are potential causes of outbreaks^14^.

Concerns regarding WSMs and WMs are underscored by other human health risks associated with them, specifically, food safety and particularly food adulteration risks, such as illegal use of antibiotics and antimicrobials^9,15,16^. This high level of food adulteration risk is exacerbated by the combination of opaque supply sources, sales of fresh and live animal products, and inconsistent supply chain conditions (e.g., cold chain), logistics and management infrastructure. The Chinese government has invested substantial regulatory resources to address challenges related to food safety and adulteration with increasing focus on WSMs and WMs, including regular testing of food^15^.

Recently, there have been voices that call for fundamental reform of WSM/WM management and even suggestions for closure of all markets^17,18^. While closing markets could alleviate zoonotic disease and food adulteration risks, this proposal ignores the central role they play. For many agricultural products, WSMs consolidate 70–80% of national supply, while WMs serve as the primary sales channel to individuals and restaurants^9,19^. Thus, eliminating these markets from the food system in China is logistically challenging and perhaps infeasible^10^. Furthermore, closing markets would adversely affect the livelihood of millions of market vendors, farmers, and transporters^10^ and create social harm. This suggests a need for practical approaches that steer regulatory resources towards riskier markets and improve management at WSMs and WMs, to mitigate public health risks they pose. It is noteworthy that a recent article published on *The Lancet Planetary Health* also argues that “safe trade and consumption of wildlife could align with existing global food safety regulations in agreement with the precautionary principle and in support of the UN Sustainable Development Goals”, which also suggests to focus on enabling smarter and targeted regulation as opposed to shutting down the entire wildlife trade^20^.

### Contributions

This paper contributes to such an approach by leveraging machine learning to generate WSM/WM market-level food adulteration risk scores from public data and demonstrating their correlation with zoonotic disease in China. In particular, the paper provides empirical support to the hypothesis that these two types of risks (i.e., food adulteration and zoonotic disease risks) are correlated, potentially because they are both affected by common system-level environmental and management deficiencies in WSMs and WMs. Moreover, this suggests that assessment of food adulteration risks can meaningfully inform the assessment of zoonotic disease risks at the market level. This observed correlation stands in contrast to the fact that zoonotic disease and food safety risks are currently regulated by different Chinese government organizations.

To accomplish this, the paper leverages a self-constructed dataset of 4.0 million public (passing and failing) records of food adulteration tests conducted by China’s state (central), provincial and prefecture-level Administrations for Market Regulation (AMRs), which are the primary organizations responsible for testing food products in WSMs/WMs^9,21–23^. This dataset integrates all tests conducted between 2014 and 2020 and posted by the national AMR, all 31 provincial AMRs, and 273 of 333 prefecture AMRs, covering nearly all major cities and important agricultural areas. Data integrity was ensured through extensive pre-processing of the raw data, including deduplication of records to avoid double counting of food safety test records, assessing the reporting rates per location with respect to the population size, and more generally checking for other potential data anomalies, such as missing values (Full details about dataset creation can be found in the Supplementary Materials and our previous paper^9^). Furthermore, this dataset is quite representative of true food adulteration risks throughout China, as the AMR agencies are legally required to publicly post all food test results on their respective websites^24^. Moreover, AMR’s policy instructs a random sampling approach within each product category, which implies that the number of tests at each market is roughly proportional to the volume of products per respective category sold in the market^25^. Finally, all AMR food safety tests conform to the same technical testing methods and standards defined in China’s national testing plan^15^.

This dataset includes 79,177 test records of animal products associated with WSMs/WMs, and an innovative unsupervised clustering methodology is developed to associate each test record with its respective market. This methodology yields a list of WSMs/WMs in China, as well as a *food adulteration risk score*, for each market, which is equal to its respective failure rate in the AMR food safety test dataset. The high-risk markets are defined as the markets with top highest 20% food adulteration risk score (equals to the failure rate of AMR testing records associated with this market) among all markets. The total volume of animal products sold through high-risk markets in each province provides *province-level risk scores*. While these risk scores are not intended to capture all nuances of food safety tests, such as adjustments for specific types of animals tested, they create significant decision support information for regulators. Notably, there previously existed no publicly available and comprehensive list of WSMs/WMs in China, let alone a method for curating all AMR tests at each market. This motivates the creation of an operational tool to communicate information to regulators, policy makers, and academics.

**Figure 1:**
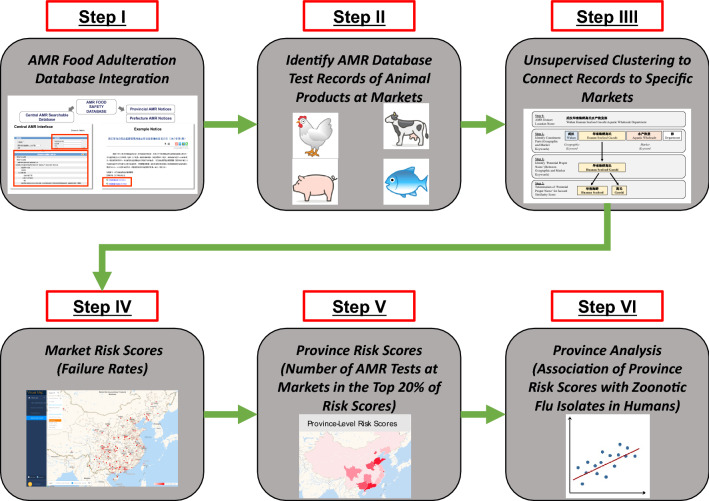
Overview of the risk score analysis to evaluate association of province food adulteration risk scores with zoonotic flu isolates found in humans. AMR dataset tests of animals are clustered to specific markets in order to create market level risk scores. These risk scores are then aggregated at the province level, and the association against province-level zoonotic flu isolates in humans is assessed. Full details can be found in the Supplementary Materials. This graph was created by Microsoft PowerPoint.

The association of food adulteration risk scores to zoonotic disease risks is validated through two analyses. First, a carefully designed linear regression indicates that province-level risk scores correlate positively with respective number of zoonotic influenza isolates (cases) in humans identified from the OpenFlu database^26^. This association holds when controlling for covariates, such as livestock production/slaughter, and distribution of markets in each province. An overview of the analysis is shown in Fig. 1. Second, specific markets associated with zoonotic outbreaks have higher risk scores than expected by chance. These analyses provide evidence that constructed risk scores give a single interpretable measure able to capture important aspects of both food adulteration and zoonotic disease risks at the market and province levels.

## Materials and methods

### Data

To assess food adulteration risk, the paper relies on a self-constructed dataset of food safety tests records conducted by China’s AMR. The AMR is the primary regulatory organization responsible for testing food products at WSMs and WMs^9,21–23^. The state/province/prefecture AMRs are also required to publicly post all food test results on their respective websites by the State Council of China^24^. Furthermore, the AMR follows a random sampling approach, meaning that the number of tests at each market is roughly proportional to the volume of products sold at the market, and that when AMR inspectors visit markets, they randomly select products for food safety testing^25^. Additionally, all AMR food safety tests conform to the methods and standards defined in China’s national testing plan, including which adulterants to test for and what legal limits define a failing test^15^. We have conducted several robustness checks and verified that there are actually slight decreases in the failure rate over time, which are statistically significant given the large amount of random sampling done by the AMR. Thus, these AMR food safety tests are comparable, and public postings offer an unbiased glimpse into food adulteration risks at various markets.

*Note:* According to the Chinese central government announcement (http://www.samr.gov.cn), the SAMR failure rate is very slowly declining over the last 5 years (2021 failure rate: 2.69%, 2020 failure rate: 2.31%, 2019 failure rate: 2.4%, 2018 failure rate: 2.42%; 2017 failure rate: 2.29%).

This AMR dataset integrates and structures 4.0 million food safety tests conducted between 2014 and 2020 and collected from postings by the state AMR, all 31 provincial AMRs, and 273 of 333 prefecture AMRs, covering nearly all major cities and important agricultural areas. This constitutes the largest and most representative set of Chinese food safety tests anywhere in the literature. See Additional Details A for more details, including data fields used in the analysis.

To map zoonotic disease outbreaks, the OpenFlu database was used to measure the number of unique zoonotic (i.e., avian and swine influenza) influenza isolates (cases) found in humans in China^26^. Limiting the OpenFlu database to human cases in China, between Jan 1, 2016 and Dec 31, 2019, there are 553 such isolates, including 366 linked to a specific province. Several other prominent zoonotic disease and influenza databases were considered for analysis, including NIH’s Influenza Virus Resources, WHO’s FluNet, and the Influenza Research Database, however other databases included either no Chinese data, insufficient geographic information for analysis (i.e., Chinese province), or insufficient data points for a statistical analysis of all provinces in China^27–29^.

*Notes*: *The OpenFluDB isolates correspond to a unique "sample of a zoonotic flu virus sequence isolated from a human patient”. Additionally, the data used in this analysis was reviewed in order to ensure each isolate also corresponded to a unique patient/case (although not necessarily a unique strain of virus).

To understand potential risks for zoonotic disease outbreaks, at markets with high food adulteration risk scores, Baidu searches (see details in Additional Details D1) are used to collect news stories related to system-level deficiencies at markets, such as illegal wild animal sales, sanitation, and lack of quarantine inspection for slaughtered animals. These deficiencies are unlikely to have direct effect on the food adulteration risk scores (i.e., AMR test failures due to adulteration).

### Market-level food adulteration risk scores

To develop market-level food adulteration risk scores, 79,177 test records of animal products (e.g., poultry, pork) related to WSMs/WMs are extracted from the AMR dataset (see Additional Details A for details).

Associating each record with a specific market requires development of a new machine learning clustering algorithm. The challenge stems from several reasons. One is the lack of a publicly available, comprehensive list of markets, requiring unsupervised clustering of ‘similar’ records (i.e., from the same market). AMR records are also inconsistent in how they specify names/addresses of the respective businesses. A third challenge is that common string distance metrics (e.g., Levenshtein distance) used by unsupervised text clustering algorithms work poorly on Chinese text^30^. More specifically, traditional machine learning methods and existing packages designed for text in Chinese (e.g., the Python package Jieba) are already widely available. However, Jieba is more focused on tokenizing sentences into vocabularies (nouns, verbs, etc.) and doesn’t work well for clustering different groups of nouns. This presents a challenge for the task of associating test records with specific market, since both the records and the market names often consist of a collection of nouns and do not constitute sentences. In particular, the accuracy obtained by existing clustering packages was well below 50%. Addressing this challenge required the development of technical approaches complementary to the existing machine learning methodologies and packages (Jieba, Jaccard similarity scores, etc.). Specifically, an entirely new machine learning tokenization approach was developed, specifically designed for this problem, allowing the application of traditional clustering algorithms.

The new tokenization methodology relies on the specific structure of market names in Chinese text. Market names follow two conventions. The first convention is: “*geographic keyword*” + “*proper name*” + “*market keyword*” (e.g., ‘Wuhan Huanan Seafood Market’, with proper name ‘Huanan’). The second convention is the same without “*proper name*” (e.g., ‘Wuhan Vegetable Wholesale Market’).

*Notes*: ‡*“geographic keyword”* means geographic location, such as province/prefecture/county name, *“proper name”* stands for the unique part of the market name representing the specific market, *“market keyword”* is a keyword indicating the record relates to a market.

**Figure 2:**
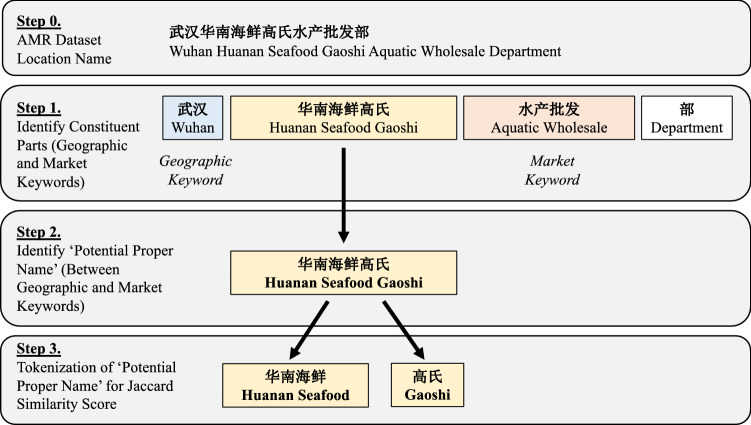
Identifying ‘potential proper names’ in AMR dataset records.

The clustering algorithm leverages this as follows. First, the *‘geographic keyword’* and *‘market keyword’* are identified through substring matching. Then, the substring between these, referred to as the *‘potential proper name’*, is extracted. If there is no such substring, the assumption is that the record relates to a market without a proper name. Figure 2 visualizes this process.

Records are clustered within each prefecture, leveraging Jaccard similarity scores, with final clusters corresponding to unique markets (see Additional Details B for full details of the Jaccard similarity score calculations)^31^. Clustering accuracy is evaluated out-of-sample for all 10,270 market-related records from Zhejiang, Hunan, and Guangdong, marking an entire cluster and all associated records are marked as incorrect if there is a single identification mistake. Finally, risk scores for each market are calculated as the failure rates of the respective AMR tests. This is done for all markets with 20+ AMR tests, to ensure robustness of the failure rates. An additional check shows that risk scores are robust to potentially biased reporting in the AMR dataset (see Additional Details C for details)*.*

### Association between risk scores and zoonotic disease risks

The paper presents three analyses to establish the association between the food adulteration risk scores and zoonotic disease related risks.

First, a linear regression model is used to evaluate the association of the food adulteration risk scores with province-level risk of zoonotic disease outbreaks. Specifically, the dependent variable is defined as the province-level number of human isolates of zoonotic flu from the OpenFlu database (see “Materials and methods” section). The key independent variable is the total province-level number of AMR tests at high-risk markets (top 20% of risk scores). Because the AMR follows a random sampling approach, this is a proxy for the volume of animals sold through high-risk markets^25^. Multiple control variables are included, most notably production/slaughter volumes by province, as well as the number of AMR tests of animals conducted at all markets in the province. Because OpenFlu isolates are related to avian/swine influenza, these variables should control against known transmission vectors. All model variables and descriptions are shown in Table 1.

**Table 1:** Predicting zoonotic outbreaks—dependent, independent, and control variables.

Variable name	Description
**Dependent variable**	
Zoonotic isolates in humans	Total unique zoonotic influenza isolates reported in humans from 2016-01-01 to 2019-12-31 in the OpenFluDB
**Independent variable**	
AMR tests at high-risk markets	Total number of tests in the AMR database conducted at markets in the top 20% of risk scores
**Control variables**	
AMR tests at all markets	Total number of tests in the AMR database conducted at markets
AMR failures at all markets	Total number of failures in the AMR database conducted at markets
AMR failure rate at all markets	Total failure rate of tests in the AMR database conducted at markets
CSY meat production	Annual meat production in the China Statistical Yearbook (2019)
CSY pork production	Annual pork production in the China Statistical Yearbook (2019)
CSY poultry production	Annual poultry production in the China Statistical Yearbook (2018)
CSY slaughtered cows	Annual slaughtered cattle in the China Statistical Yearbook (2018)
CSY slaughtered pigs	Annual slaughtered pigs in the China Statistical Yearbook (2018)
CSY slaughtered poultry	Annual slaughtered poultry in the China Statistical Yearbook (2018)
CSY slaughtered sheep	Annual slaughtered sheep and goats in the China Statistical Yearbook (2018)
Average daily high temperature	From the China Meteorological Data Service Center (https://data.cma.cn/en)

The China Statistical Yearbook data is taken from the last available year (http://www.stats.gov.cn/english/).

Linear regression models are estimated in-sample using variables from Table 1. First, best-fit model is identified minimizing Akaike information criterion (AIC) across all combinations of variables. The benchmark model is identified likewise but excluding the key independent variable. AIC difference is compared, to heuristically evaluate model improvement^32^. See Additional Details E for details related to linear regression data assumptions.

Second, risk scores of all specific markets associated with zoonotic diseases outbreaks are considered. Under the null hypothesis (i.e., market risk scores are not associated with outbreaks), the market risk score percentiles for these markets distribute uniformly between 0 and 100%. The probability (one-sided test) that the two markets are in the top *x*% of risk scores, under the null hypothesis, is $p={\left(\frac{x}{100}\right)}^{2}$.

Third, the paper evaluates association of market risk scores with existence of negative news related to system-level environmental and management deficiencies that are risk factors for zoonotic disease outbreaks (e.g., allowing sale of illegal wild animals—see Sect. 2a). The 40 highest-risk markets are each paired with a lower-risk market, from the same geography and with approximately the same number of AMR tests (see details in Additional Details D2). This results in 31 market pairs within the same prefecture, and the remaining within the same province. The 40 highest-risk markets have 2094 corresponding AMR tests, while low-risk markets have 2095 tests. Under the null hypothesis (i.e., market risk scores are not associated with incidence of news stories), the number of high-risk and low-risk markets with such news stories would respectively be drawn from an i.i.d. binomial distribution. Proportions can then be compared with a two-sample binomial test, to evaluate whether high-risk markets are more likely to have such negative news (one-sided test).

## Results

### Clustering results

The results of the clustering algorithm (assigning AMR records to specific markets) are shown in Table 2 below. Note that the 853 high-volume markets (i.e., 20+ AMR tests) cover 54% of all tests at markets and we chose those markets for our analysis. Because large markets consolidate 70–80% of the supply for most agricultural products, it follows from Table 2 that high-volume markets distribute a substantial amount of the animal supply.

**Table 2:** Clustering results: number of markets identified and distribution of tests per market.

Number of tests per market (n)	Number of markets with n+ tests	Percent of markets covered (%)	Total number of tests at all markets with n+ tests	Percent of tests covered (%)	Average risk score
≥ 100	60	0.6	13,405	17.0	0.0435
** ≥ 20**	**853**	**9.1**	**42,664**	**54.0**	**0.0367**
≥ 10	1908	20.4	56,766	71.8	0.0371
≥ 5	3533	37.8	67,544	85.5	0.0409
≥ 2	6818	72.8	76,481	96.8	0.0468
≥ 1	9354	100.0	79,022	100.0	0.0500

The first column represents different inclusion thresholds for markets to be included in the analysis as per the number of tests associated with the respective market. The 2nd to 5th columns provide statistics on the markets included in the group: number of markets in the group, the fraction these markets out of all markets, the total number of tests associated with markets in the group, the fraction these tests out of all tests and the average risk score of the markets in the group. For example, if we choose markets that have at least 100 tests (first row), then only 0.6% of all markets and 17% of all tests will be covered in our analysis.

Selected markets are in bold.

Based on manual review, the out-of-sample clustering accuracy for all 10,270 market-related records from Zhejiang, Hunan, and Guangdong provinces is between 89.02 and 93.44% at the record-level, and 91.7% to 94.9% at the cluster-level (see Supplementary Materials for additional details).

**Figure 3:**
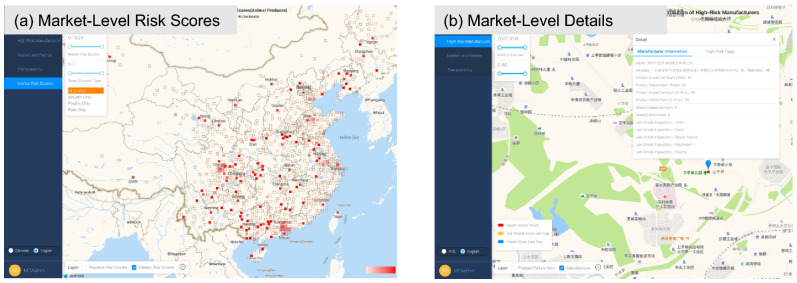
Market risk score tool. The market-level risk scores are shown in (**a**) with darker (lighter) red indicates a higher (lower) value of risk scores. When clicking on a market, the operational tool also communicates the name and address as well as the results of AMR food safety tests in that specific market as shown in (**b**), including what types of food were tested (e.g., poultry, pork), and what the results were for these food adulteration tests. The operational tool (https://foodsafetyinchina.azurewebsites.net/) was created in collaboration with iSoftStone using AntV G2 (https://g2.antv.vision/en).

After clustering, markets are assigned food adulteration risk scores equal to their respective failure rates in the AMR database. These risk scores were used to create an operational tool for regulators, communicating the results of this market risk score analysis. See Fig. 3 below for an overview of the market risk score tool.

### Association between provincial food adulteration risk scores and zoonotic disease risk

**Figure 4:**
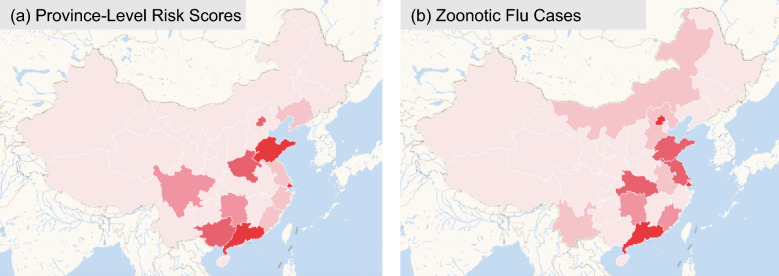
Association of risk scores and zoonotic disease by province. The province-level risk scores are shown in (**a**) and the number of zoonotic flu isolates (cases) in humans per province is shown in (**b**). In both maps, darker (lighter) red indicates a higher (lower) value of risk scores and zoonotic flu isolates in humans. The correlation between these variables is 0.701, implying that the province-level risk scores explain nearly 50% of the variation in the number of zoonotic flu isolates in humans (interpreting the coefficient of determination). This map was created by the software provided by Dituhui: https://www.dituhui.com/.

The correlation between province-level risk scores and respective zoonotic flu isolates (cases) in humans is shown in Fig. 4.

A linear regression model is used to evaluate association of province-level risk scores with zoonotic influenza cases in humans, while controlling for covariates, such as livestock production, slaughter, and the distribution of markets in each province. All model variables and descriptions and the best-fit linear regression model are shown in Tables 1 and 3, respectively.

**Table 3:** Linear regression—model coefficients.

Variable name	Coefficient estimate (std dev)	p-value
(Intercept)	8.34767 (5.20337)	0.12
AMR tests at high-risk markets	0.04321 (0.00762)***	< 1 * 10^–5^
CSY meat production	− 0.02801 (0.01753)	0.12
R-squared	0.534	
AIC	268.59	
No. observations	31	

Standard errors are reported in parentheses. *, *** indicates significance at the 90% and 99% level, respectively.

The best-fit model achieves an AIC of 268.59, and the benchmark model (only using control variables) an AIC of 275.33. This AIC improvement exceeds the common heuristic of 2.0 used to show model improvement^32^. The in-sample coefficient of determination (R^2^) improves from 0.421 to 0.534 with the addition of the key independent variable (number of AMR tests at high-risk markets). Furthermore, as observed in Table 3, the model coefficient for AMR tests at high-risk markets is significant (p < 1 * 10^–5^).

### Risk scores at specific markets implicated in spreading zoonotic disease

Zoonotic disease outbreaks have been tied to specific markets twice per literature review. Specifically, the *Wuhan Huanan Seafood Market*, associated with the first COVID-19 cluster, is among the top 6% of risky markets^11^. Likewise, the *Beijing Xinfadi Seafood Market*, linked to a secondary COVID-19 spread after 57 days of no new cases in Beijing, is among the top 21% of risky markets^12,33^. The probability of both markets being in the top 21% of risk scores is statistically significant (p = 0.042) rejecting the null hypothesis that the risk scores are not correlated with zoonotic disease risk.

### Published media news related to high-risk markets

Finally, the association of market risk scores with news related to system-level environmental and management deficiencies was analyzed, through a paired analysis of the 40 highest risk markets, and 40 lower risk markets normalized on geography and market size. Negative news was found for 27 out of 40 high-risk markets and 13 of 40 lower-risk markets, indicating statistical significance (two sample binomial test, p = 0.0017). Notably, two of the high-risk markets had news stories related to illegal sale of wild animals, which is especially risky for zoonotic disease.

## Discussion and conclusion

While many researchers have noted the link between Chinese WSMs/WMs and zoonotic disease outbreaks, this has not led to many actionable recommendations^1–7,13^. In fact, the only such recommendation identified in the literature was calls for closure of all WSMs and WMs^17,18^. However, this approach lacks practicality and would have serious secondary consequences because of the centrality of these markets in China’s food system. As noted in the introduction, these markets are major distribution points in the Chinese agricultural supply chain, consolidating 70–80% of supply for many agricultural products^9,10,19^. Closing all markets would adversely affect the livelihood of millions of market vendors, farmers, and transporters^10^.

Addressing an important need, this research provides an important foundation to a new and more targeted risk management approach to address zoonotic disease and food adulteration risks posed by WSMs and WMs^10^. Specifically, the paper develops individual market-level food adulteration risk scores based on publicly posted AMR data. Empirical analyses show that these risk scores correlate with zoonotic disease risks. First, it is shown that province-level risk scores are significantly associated with the respective number of zoonotic flu isolates identified in the province. It is also shown that market-level risk scores are significantly higher at markets associated with zoonotic disease outbreaks. These analyses support the hypothesis that food adulteration and zoonotic disease related risks are driven, at least partially, by common system-level environmental and management deficiencies in WSMs and WMs. A media news analysis provides additional evidence that markets with high risk-scores are significantly more likely to have negative news related to sanitation or management deficiencies that directly drive zoonotic disease risk (e.g., allowing the illegal sales of wild animals), further supporting the hypothesis.

The methodology developed in this paper is also aligned with China’s current policy approach to food safety management and regulation. In recent years, the Chinese government has invested substantial efforts into improving food safety, notably by publishing food inspection results, and increasing transparency for both public complaints about food safety and media reporting, all with the goal of increasing access to relevant information^24,34^. The analyses in this paper illustrate how this data could enable market-level risk assessment. China has already acknowledged the strategic importance of WSMs and WMs and has emphasized the need for increased monitoring and inspections of markets^15^. Therefore, the risk-based methodology in this paper may support this effort by allowing the Chinese government to pursue more targeted policies that focus on high-risk markets and lower both food adulteration risk and zoonotic disease risk. The ability to better identify risk at the local (market) level is particularly critical given the size of the food supply chain in China and the decentralized regulatory environment. Another important insight that emerges from the analyses in the paper is that food safety and zoonotic disease risks should regulated in integrated manner.

The work in this paper is not only a first-of-its-kind approach to use advanced data analytics for the identification of specific risky supply chain nodes (markets), but it also provides empirical evidence for the hypothesis that food adulteration and zoonotic diseases risks share common drivers, which can directly inform public health policies. While more research is needed to better understand this association and identify the specific risk drivers, this approach has the potential to offer entirely new ways of understanding why zoonotic disease outbreaks occur, as well as complementary regulatory approaches to reducing pandemic risks.

## Supplementary Information

Supplementary Information.

## Data Availability

All data, code, and materials used in the analysis are available upon request by the corresponding author. The datasets generated during and analyzed during the current study are available from the corresponding author on reasonable request.

## Code availability

The code used for the analysis is also available from the corresponding author on reasonable request.
