# Supplementary material for: Leveraging machine learning to assess market-level food safety and zoonotic disease risks in China
Source: Sci Rep. 2022 Dec 15;12:21650. doi: 10.1038/s41598-022-25817-8 (PMC9755119; doi:10.1038/s41598-022-25817-8)
Supplement: Supplementary file 1 — Supplementary Information. [file 41598_2022_25817_MOESM1_ESM.docx]

Supplementary Materials for

Leveraging Machine Learning to Assess Market-level Food Safety and Zoonotic Disease Risks in China

Qihua Gao, Retsef Levi, Nicholas Renegar

Correspondence to: [retsef@mit.edu](mailto:retsef@mit.edu)

**This PDF file includes:**

Supplementary Text

Tables S1 to S9

Supplementary Text

# Additional Details A. AMR Dataset

**A1. AMR Dataset Overview:**

To assess food adulteration risk, the paper relies on a self-constructed dataset of food safety tests records conducted across China by the AMR. The AMR is one of two Chinese government agencies responsible for regulating food safety (together with the Ministry of Agriculture. This dataset integrates and structures 4.0 million food safety tests conducted between 2014-2020 and posted by the state AMR, all 31 provincial AMRs, and 273 of 333 prefecture AMRs, covering nearly all major cities and important agricultural areas. Full dataset details are described in Jin et al. (2020).^9^ See Table S1 for data fields used in the analysis.

**Table S1. AMR Dataset Fields**

**A2. Adjudicating Product Category for Unlabeled Records:**

To create the market risk scores, the AMR dataset was filtered to tests of animals (poultry, pork, aquatic products, etc.) This involves several analytics techniques, including the use of a neural network to adjudicate product categories for unlabeled data Full details of the dataset construction including this adjudication process for product category are described in Jin et al. (2020).^9^

For more than 99% of the food safety tests, a detailed food name is reported (e.g., "500g fresh blackfish"), and for about 80% of the food safety tests a product category is reported. However, the product categories are not on a consistent basis. There are two main standards for product categories: AMR product standards, and the Chinese Guobiao (GB) standards (http://www.gbstandards.org). In the publicly posted AMR food safety tests, both sets of standards commonly appear, as well as a small number of product category names meeting neither set of standards. Overall, there were more than 400 unique product categories reported by inspectors.

The first step to create the product category classification, is to take the labeled data, for which some product category is reported, and remap to a consistent classification scheme. Product categories were mapped together, maintaining the most granular detail possible, consistent with the AMR and GB standards. For instance, the product categories of (aquatic products, fish, shrimp, meat, pork, poultry), would map to (aquatic products, meat). Completing this process for all 400 unique product categories in the raw data, created 29 product categories in the final mapping.

For the 20% of the data where the product category was not reported, a neural network was trained on the labeled data to predict the product category. Food names were translated to English to introduce tokenization, with the Google Cloud Translation API (https://cloud.google.com/translate/), and bag-of-words was used to collect the 1,000 most common sets of tokens in the detailed food name (including single tokens and bigrams). The supply chain type of the organization being tested (e.g., restaurant) was also used as an input to the neural network. For the neural network, the product category was predicted using a ‘softmax’ function in the final layer. This output assigns a probability to each of the 29 product categories.

To evaluate the neural network, a 60/20/20 split was used to train, validate, and test on the labeled data. A neural network with three layers, 75 nodes per layer, and no dropout was selected using the validation data. A threshold was added, to only assign product category when the neural network assessed a probability of at least 70% for its prediction. This was added based on a manual review to avoid mistakes where no terms from bag-of-word appeared in the food name, and for situations where the product category was too ambiguous. On the test dataset, the neural network predicted the product category correctly 76.3% of the time, incorrectly 6.9% of the time, and left the product category unlabeled 17.2% of the time. A manual review of 100 of the incorrect guesses showed that about one-third of these were for ambiguous food names, where the predicted product category and the actual label were both reasonable. For another one-third of these, the predicted category was correct while the inspector made an error in labeling the product category. Therefore, the neural network has comparable results to human labeling.

This neural network was then applied to all records in the data for which product category was unlabeled. Because these food names did not appear materially different to food names in the labeled data, a similarly high level of accuracy is expected. A manual review of 100 randomly selected predictions for this unlabeled data was also conducted.

**A3. Identifying Records Collected or Sourced from Markets:**

To identify AMR dataset testing records that can be linked to a wholesale or wet market, a keyword dictionary is used to determine whether business names correspond to a market. This “market keywords” dictionary is developed by analyzing the patterns of 200 different market names in China. For example, if a test was collected from the Wuhan Huanan Seafood Market, then the pair of terms ‘Seafood Market’ shows that this test is related to a wholesale or wet market.

Because market names usually consist of terms that mean “market” and terms that mean different types of “food”, the “market keyword” dictionary is created by three steps: 1. Building two sets of “strings” that can be used for markets’ names, one from common substrings of “market” names and one from unique words/characters of different types of “food”. 2. Use different arrangements and combinations of those "strings" to create the first iteration of keywords dictionary. 3. Validation of the keywords by querying the AMR dataset and manually reviewing a total of 1,000 randomly selected records that matched these keywords. The keywords are adjusted by adding and deleting strings to maximize accuracy, and the process is iterated until the validation step yields accurate results. A final version of the keywords dictionary was created after 5 iterations. The keywords are also cross-referenced with keywords from a list of ~700 WSMs published by the MOA and confirmed that the naming methods indeed align with our keyword dictionary.

The full list of keywords used to identify markets were: 菜市场, 大市场, 综合大市场, 街市, 果蔬大市场, 果蔬交易, 果蔬批发, 果蔬市场, 果蔬城, 果蔬物流, 果蔬综合市场, 集贸市场, 集贸大市场, 集贸综合市场, 农产品大市场, 农产品综合市场, 农产品交易, 农产品批发, 农产品市场, 农产品物流, 农批, 农副产品大市场, 农副产品交易, 农副产品批发, 农副产品市场, 农副产品物流, 农副产品综合市场, 批发市场, 市场, 农汇, 农贸城, 农贸大市场, 农贸市场, 食品城, 食品交易, 食品批发, 食品交易, 食品物流, 食品大市场, 食品综合市场, 蔬菜大市场, 蔬菜果品批发, 蔬菜批发, 蔬菜市场, 蔬菜城, 蔬菜物流, 蔬菜综合市场, 水产市场, 水产交易, 水产批发, 水产批售, 水产物流, 水产中心, 水产城, 水产世界, 水产大世界, 水产大市场, 水产品批发, 水产品批售, 水产品物流, 水产品市场, 水产品交易, 水产品中心, 水产品城, 水产品世界, 水产品大世界, 水产品大市场, 水产品综合市场, 水产综合市场, 海鲜市场, 海鲜批发, 海鲜批售, 海鲜物流, 海鲜交易, 海鲜中心, 海鲜城, 海鲜世界, 海鲜大世界, 海鲜大市场, 海产批发, 海产批售, 海产物流, 海产交易, 海产市场, 海产中心, 海产城, 海产世界, 海产大世界, 海产大市场, 海产品批发, 海产品批售, 海产品物流, 海产品交易, 海产品市场, 海产品中心, 海产品世界, 海产品大世界, 海产品大市场, 海产品综合市场, 海鲜综合市场, 海产综合市场

This keyword dictionary was also assessed out-of-sample on all 10,270 records with a market keyword from Zhejiang, Hunan and Guangdong provinces, to ensure that the analysis in this paper only includes data related to markets. Specifically, for all 10,270 records, the name/address of the markets were searched on Google and Baidu to verify that the business is in fact a wet market or wholesale market, and not some other type of business. These checks found almost no evidence of mistakes. For only 0.00%, 0.00%, and 0.08% of the records in Zhejiang, Hunan, and Guangdong provinces, respectively, the business was found to be misclassified and related to a business other than a market. Likewise, for 90.75%, 88.03%, and 93.52% of the records in Zhejiang, Hunan, and Guangdong provinces, respectively, it was verified that this record does relate to a market. For the remaining records, the name/address searched on Google and Baidu did not yield any results related to the business. Table S2 below shows the results of these checks.

**Table S2: Accuracy of Identifying AMR Dataset Tests Related to Markets**

# Additional Details B. Clustering Methodology

This section provides full details of the clustering methodology (see Section 2c). This clustering is challenging, and requires development of a new algorithm, for several reasons. One is the lack of a publicly available, comprehensive list of markets, requiring unsupervised clustering of ‘similar’ records (i.e., from the same market).

AMR records are also inconsistent in how they specify names/addresses of the respective businesses (see Table S3 for examples). A third challenge is that common string distance metrics (e.g., Levenshtein distance) used by unsupervised clustering algorithms work poorly on Chinese texts, particularly for highly inconsistent business names.

**Table S3: AMR Dataset: Different Expressions of “Wuhan Huanan Seafood Wholesale Market”**

Additional Details B1 includes a high-level technical overview of the clustering method. Additional Details B2 includes a description of how market keywords and geographic keywords were identified in AMR records. Additional Details B3 describes the calculation of Jaccard similarity scores on ‘potential proper names’ of markets. Additional Details B4 describes the clustering methodology, based on the Jaccard scores. Additional Details B5 describes details of the out-of-sample accuracy evaluation for the clustering methodology.

### B1. Overview of Clustering Methodology and Results

The unsupervised clustering methodology developed and used in this paper, relies on the specific structure of Chinese market names. In particular, a review of all aquatic wholesale markets in Zhejiang, Guangdong and Hunan provinces, and discussions with academic experts, reveals markets in China adhere to two naming conventions. Roughly half the markets in China have a ‘proper name’ specific to that market (e.g., Wuhan Huanan Seafood Market has proper name ‘Huanan’), and follow the format: “*geographic keyword*” + “*proper name*” + “*market keyword*”. Other markets do not have a “*proper name*” and such markets follow the format: “*geographic keyword*” + “*market keyword*” (e.g., Wuhan Vegetable Wholesale Market). A broad review of market-related records in the AMR dataset confirms these structures.

*Notes. “geographic keyword”* means geographic location information, such as province/prefecture/county name, *“proper name”* stands for the unique part of the market name that only represents a specific market, *“market keyword”* is one of the keywords that indicates this record is related to a market.

The clustering methodology leverages this structure as follows. First, the *‘geographic keywords’* and *‘market keywords’* are identified through string matching (Additional Details B includes more details). Then, the methodology extracts the substring between the geographic and market keywords, which is referred to as the *‘potential proper name’*. If there is no such string, the assumption is that the record relates to a market without a proper name. For records with *‘potential proper name’* strings, the methodology then ‘tokenizes’ the string into constituent Chinese words using natural language processing (NLP) via the Python package “Jieba” (Jieba python package: https://github.com/fxsjy/jieba) This is necessary as Chinese text lacks natural space between words and divisions are implicit.

The methodology then clusters all records in each prefecture in two steps. First, it clusters records without a proper name, based on the unique pairs of ‘geographic keywords’ and ‘market keywords’, as these terms uniquely describe the market names when proper names are absent. Second, the ‘potential proper names’ are clustered together. This is still difficult, as AMR inspectors enter data by hand, and the proper name of the record could still have many variations and abbreviations for the market, thus requiring a robust clustering method. To cluster, Jaccard similarity scores are computed between all pairs of identified ‘potential proper names’, first treating all characters in the ‘potential proper names’ as unique elements, and second treating all tokens as unique elements ^21^. Then, ‘potential proper name’ pairs with Jaccard similarity scores above a threshold (0.4) are clustered together, where the threshold is optimized over a training set of 10 prefectures. Full details of the clustering methodology can be found in Additional Details B.

Finally, the clustering algorithm and accuracy is evaluated manually, out-of-sample for all 10,270 market-related records from Zhejiang, Hunan, and Guangdong provinces. A rigorous metric is used for this evaluation, where an entire cluster and all associated records are marked as incorrectly clustered if there is a single mistake (e.g., if a cluster has 49 records from market A and 1 from market B, then all 50 records are labeled ‘incorrect’). Even with these rigorous evaluation metrics, the clustering algorithm achieves accuracy of 89.02% to 93.44% at the record-level, and 91.7%, to 94.9% at the cluster-level across the three provinces. More details about the accuracy evaluation are given in Additional Details B. Clustering results are shown in Table 2, in the main paper.

### B2. Identifying Geographic Keywords and Market Keywords in AMR Records

To isolate a *‘potential proper name’* for the market within an AMR record (see Section 2c), the *‘geographical keyword’* and *‘market keyword’* are first identified in the record’s in the location name. The ‘potential proper name’ is then equal to any string between these keywords.

To identify the ‘geographical keyword’, the methodology first searches the locations for any substrings within an enumerated list of all provinces, prefectures, counties, districts, and villages in China. In the absence of any of these substrings, the algorithm instead searches for Chinese character related to a geographical area: (省(province), 市(prefecture), 县(county), 区(district), 镇(town), 村(village)). In order to identify the ‘market keyword’, the methodology searches the locations for any of the market keyword substrings that are listed in Additional Details A2. Because this analysis is only for AMR dataset records that contain one of these market-related substrings (see Additional Details A2), such a keyword always exists in the record.

### B3. Calculating Jaccard Similarity Scores

Because AMR inspectors enter data by hand, the ‘potential proper name’ of the records, as identified in Additional Details B2, could still have many variations and abbreviations for the market. For example, as shown in Table 2, records belonging to the same market can be recorded very differently in the AMR dataset. Therefore, a metric is needed for comparing two ‘potential proper names’ to check if they refer to the same market. The primary similarity metric used in this paper is the Jaccard similarity score, which is a measure for two sets that assesses the relative intersection of elements in those two sets. ^21^ Formally, for two sets A and B, the Jaccard similarity score is given by $\frac{|A\cap B|}{|A| + |B| - |A\cap B|}$.

In particular, two ‘potential proper names’ are assigned two Jaccard similarity scores. The first is computed by treating the unique characters as elements, and the second by treating the tokens (tokenization with “Jieba”) as the elements. For instance, if the two ‘potential proper names’ were “ABCDEF” and “EF”, with tokenization {“ABCD”, “EF”} and {“EF”}, then the Jaccard similarity score treating each character as an element would be ⅓, and the Jaccard similarity score treating each token as an element would be ½. The similarity metric used in this paper is the maximum of these two scores.

### B4. Clustering ‘Potential Proper Names’

The clustering methodology relies on the Jaccard similarity scores introduced in Additional Details B3, as well as a heuristic rule. Specifically, two ‘potential proper names’ are clustered together if either of the following two conditions hold: 1) the maximum Jaccard similarity score (treating the characters or the tokens as elements, see Additional Details B3) are above a threshold *t_j_*, 2) one ‘potential proper name’ is a substring of the second ‘potential proper name’, but is not at the beginning of the second ‘potential proper name’. For instance, this second heuristic rule would cluster “ABCDEF” and “EF”, but not “ABCDEF” and “A”. This heuristic rule is designed to cluster records from the same market where one record has a very long street name within its ‘potential proper name’ and the other does not.

The above clustering of ‘potential proper names’ is done sequentially. In particular, if any two records are clustered together, then any larger clusters they are part of are also combined. For example, if ‘potential proper names’ A and B are clustered together in the first step, and then ‘potential proper names’ A and C are clustered together in the second step, the cluster will contain all three ‘potential proper names’ [A, B, C]. Merging is complete when all pairs of ‘potential proper names’ that meet either clustering criteria above (Jaccard similarity score above threshold *t_j_*, or the heuristic rule), belong to the same cluster. It should be noted that this sequential clustering is order invariant (i.e., the final clusters will be the same, regardless of which order pairs of ‘potential proper names’ are clustered).

Finally, within each cluster, county level information is considered (if it is available in the AMR dataset, around 50% of records have county information). If two ‘potential proper names’ have records with different counties, the cluster is divided based on county. This is to separate two markets that have the same proper name but in fact are branch offices of the same parent company sharing the same brand/company name.

For this clustering methodology, both the threshold *t_j_*, and the heuristic rules were developed using a training set of 10 prefectures with a total of ~10k records. The training set suggests that an empirical threshold of *t_j_*=0.4 works best for clustering. Tables S4 and S5 below give some examples of the types of false positives that commonly happen in clustering. Tables S6 and S7 give some examples of false negatives.

**Table S4: False Positives in Clustering – Example One**

**Table S5: False Positives in Clustering – Example Two**

**Table S6: False Negatives in Clustering – Example One**

**Table S7: False Negatives in Clustering – Example Two**

### B5. Clustering Accuracy

The accuracy of the clustering algorithm is then evaluated out-of-sample, by manually assessing correctness for all 10,270 records from Zhejiang, Hunan and Guangdong provinces. Specifically, the clusters are ordered, and then each cluster is examined sequentially. For each cluster, it is labeled as inaccurate based on either of the following conditions: 1. If any record in the cluster belongs to the same market as a previous cluster; 2. If two records in the cluster belong to different markets. If a cluster is labeled as inaccurate, then all records in the cluster are also labeled as inaccurate. The resulting accuracy rates for Zhejiang, Hunan, and Guangdong provinces are 89.02%, 93.44%, and 91.97% at the record level and 92.4%, 94.9%, and 91.7% at the cluster level, respectively. More details are described in Table S8.

**Table S8: Results of Clustering Accuracy Checks**

# Additional Details C. Risk Score Adjustments

One potential issue in calculating market risk scores directly from overall failure rates, is that reporting bias might impact results. While AMR organizations are legally required to publicly post all tests (see Section 2a), they could hypothetically fail to do so because of a lack of oversight. To account for this, the following robustness check is performed. The underlying assumption is that all levels of AMR organizations (state/central, provincial, prefecture) should achieve similar aggregate failure rates when conducting tests in the same prefecture, and any differences in failure rates are strictly due to underreporting of either passing or failing test results. For example, Table S9 below shows the failure rates by data source type for tests conducted in Beijing.

**Table S9: Failure Rates by Data Source Type for AMR Dataset Tests in Beijing**

Then, for each market, an adjusted failure rate (risk score) is calculated using Bayes’ rule, based on an assumed level of underreporting 23. Finally, these adjusted risk scores are compared to the unadjusted risk scores (raw failure rates for each market in the AMR dataset).

**C1. Description of Approach**

The goal of this section is to adjust market failure rates for reporting bias, by estimating a reasonable *baseline* failure rate for each prefecture, and then making an assumption about the level of under-reporting of *passing* or *failing* test results for each data source in that prefecture. The baseline failure rate is intended to be an estimate of the true failure rate observed in a prefecture, if a data source were to report all test results. For example, in Table S9, it would be assumed that the true baseline failure rate of tests done in this prefecture is somewhere around 1%, with the state-level AMR slightly underreporting failed tests, and the province-level AMR slightly underreporting passing tests.

To make this explicit, the baseline failure rate, denoted $f_{baseline}$, is set equal to the geometric mean of the three failure rates as shown in Table S9. This estimate is motivated by a review of the data. Typically, in each prefecture, the three data source types either have relatively similar failure rates, or a single outlier. In these cases, the geometric mean yields a baseline close to the failure rates of data sources that are consistent with each other.

**C2. Calculating Adjusted Market Risk Scores When** $f_{data}> f_{baseline}$

If the failure rate of a data source, denoted $f_{data}$, is greater than the baseline failure rate, then the proportion of passing tests $p_{pass}$being reported can be deduced. Assuming that the true failure rate of all tests from the data source (including unreported tests) is $f_{baseline}$, and assuming that all failing tests are reported, it follows that:

$$f_{data}=\frac{f_{baseline}}{f_{baseline}+ p_{pass}*(1-f_{baseline})}.$$

Which implies that:

$p_{pass}=\frac{f_{baseline} *(1-f_{data})}{f_{data}*(1-f_{baseline})}$ (1)

Assuming that any passing test is equally likely to be reported, Bayes rule can be used to determine the expected failure rate of a market 18. We let $n_{rp}$ ($n_{rf}$) denote the number of passing (failing) tests related to the market and reported in the data. We again let $f_{data}$ denote the average failure rate of the data sources reporting the data, and $f_{baseline}$ the corresponding baseline failure rate for the prefecture. Assuming a uniform prior on the total number of passing tests done at the market $n_{pass}$, the probability of $n$ such tests, including unreported tests, is given by:

$\mathcal{P}\left( n_{pass}=n|n_{rp}, p_{pass} \right)= \frac{\mathcal{P}\left( {Bin(n,p}_{pass})=n_{rp} \right)}{\sum_{i=n_{rp}}^{\infty} \mathcal{P}\left( {Bin(i,p}_{pass})=n_{rp} \right)}$ (2)

Where $Bin(n, p)$denotes the binomial probability distribution. The expected adjusted failure rate of the market is then:

$E\left[ Adjusted Failure Rate \right]= \sum_{n=n_{rp}}^{\infty} \frac{n_{rf}}{n+n_{rf}}\mathcal{*P}\left( n_{pass}=n|n_{rp}, p_{pass} \right)$ (3)

Where the probabilities are given as in Equation 2 above.

Example 1

As an example, consider the following hypothetical market. Let $n_{rp}=5$ and $n_{rf}=16$, so the unadjusted market failure rate is $\frac{5}{5+16}=76.2\%$. Furthermore, let $f_{data}=25.12\%$, and $f_{baseline}=2.63\%$.

From Equation 1, $p_{pass}=\frac{0.0263*(1-0.2512)}{0.2512*(1-0.0263)}=0.081$

From Equations 2 and 3, the risk score (expected adjusted failure rate) for the market is:

$$E\left[ Adjusted Failure Rate \right]= \sum_{n=5}^{\infty} \frac{16}{n+16}*\frac{\mathcal{P(}Bin\left( n,0.081 \right)=5)}{\sum_{i=5}^{\infty} \mathcal{P(}Bin\left( i,0.081 \right)=5)}=19.88\%$$

**C3. Market Risk Scores When** $f_{data}< f_{baseline}$

Likewise, if $f_{data}< f_{baseline}$, and assuming that the true failure rate of all tests from the data source (including unreported tests) is $f_{baseline}$, and assuming that all passing tests are reported, this implies the proportion of failing tests $p_{fail}$ being posted satisfies:

$$f_{data}=\frac{{p_{fail}*f}_{baseline}}{{p_{fail}*f}_{baseline}+ (1-f_{baseline})}.$$

Which implies that:

$p_{fail}=\frac{f_{data} *(1-f_{baseline})}{f_{baseline}*(1-f_{data})}$ (4)

Assuming that any failing test is equally likely to be reported, and assuming a uniform prior on the total number of failing tests done at the market $n_{fail}$, the probability of $n$ such tests, including unreported tests, is given by:

$\mathcal{P}\left( n_{fail}=n|n_{rf}, p_{fail} \right)= \frac{\mathcal{P}\left( {Bin(n,p}_{fail})=n_{rf} \right)}{\sum_{i=n_{rf}}^{\infty} \mathcal{P}\left( {Bin(i,p}_{fail})=n_{rf} \right)}$ (5)

Where $Bin(n, p)$denotes the binomial probability distribution. The expected adjusted failure rate of the market is then:

$E\left[ Adjusted Failure Rate \right]= \sum_{n=n_{rf}}^{\infty} \frac{n}{n+n_{rp}}\mathcal{*P}\left( n_{fail}=n|n_{rf}, p_{fail} \right)$ (6)

Where the probabilities are given as in Equation 5 above.

**C4. Risk Score Adjustment Results**

After the risk score adjustments, the market-level risk scores remain quite similar. The correlation between the adjusted and unadjusted risk scores is 0.919. Additionally, 82 of the top 100 highest-risk markets (based on unadjusted risk scores) remain in the top 100 following the adjustments. Therefore, the risk scores seem very robust to any potential bias in the AMR dataset, and the rest of the paper analyzes results for the unadjusted risk scores.

# Additional Details D. Published News Related to Markets

This section describes the searching methodology, pairing methodology, as well as several insights and findings related to the news stories found for the high-risk and low-risk markets, as described in Section 3c.

**D1. Searching Methodology**

News stories related to system-level environmental and management deficiencies were identified for each market, by manually searching Baidu news (Baidu Advanced Search is used by clicking “setting” on the top right corner of Baidu webpage, and selecting “advanced search”). Specifically, the Baidu advanced search feature was utilized, by using the market name as the primary keyword, and for each of the following secondary keywords: sanitary, remediation, inspection, complaint, and food safety. From these search results, negative news stories were manually identified related to system-level deficiencies described above.

**D2. Pairing Methodology**

The 40 highest-risk markets were each paired with a low-risk market, controlling for geographic area and the number of tests per market in the AMR database, as these features might also affect the likelihood of negative news being published. First, all list of other markets from the same prefecture (not in the top 40 highest risk scores) are obtained from the clustering result. Second, all list of other markets from the same province, if none are in the same prefecture (not in the top 40 highest risk scores) are obtained from the clustering result. Finally, the lowest risk markets (lowest failure rate) are paired with roughly the same number of tests from the above group. After this pairing, 31 of the 40 low-risk markets were in the same prefecture as their paired high-risk market, while the other 9 were in the same province. The group of 40 high-risk markets had 2,094 corresponding tests in the AMR database, while the low-risk markets had 2,095 tests.

**D3. Insights and findings**

Several findings about negatives news stories are summarized based on manual review. First, negative news stories were published by organizations from the following main categories: 1. government websites; 2. newspapers (in particular undercover journalists’ investigations); 3. internet forums established years before. Second, most of the negative news was reported between 2011 and 2016. News stories published after 2016 tend to be more positive, potentially related to either improvements in food safety or increased internet censorship in China. Finally, it should be noted that some wet and wholesale markets, especially the high-risk ones, include ‘road market’ sections that have vendors in booths on the roads surrounding the markets. Anecdotally, these markets appear especially prone to news stories regarding poor sanitation conditions.

# Additional Details E. Validation of Linear Regression Assumptions

Linear regression data assumptions were reviewed prior to running the analysis. Normality assumptions (of the residuals) were found to hold in general, other than a few data points.

**Table S1 – S9**

**Table S1. AMR Dataset Fields**

| **AMR Dataset Fields and Descriptions** | |
| --- | --- |
| **Field** | **Description** |
| Food Name | Detailed name of food being sampled (e.g., blackfish) |
| Product Category | Higher-level category for the food being sampled (e.g., aquatic products) |
| Sampled Location Business | Name and address of business where AMR inspectors collected the food being sampled |
| Source Location Business | Name and address of the upstream business in the supply chain where the product was sourced from (e.g., food collected by AMR inspectors from a supermarket/restaurant could have been sourced from a WSM, in which case the WSM is the ‘source location’) |
| Test Outcome | Passing or failing (based on whether the food being sampled is within the legal adulterant standards for that product category, as defined by the AMR) |

**Table S2: Accuracy of Identifying AMR Dataset Tests Related to Markets**

| **Province** | **Total Records** | **Verified as Relating to a Market (Correctly Classified)** | **Verified as Not Relating to a Market (Incorrectly Classified)** | **Unverified (No Information Found for the Business)** |
| --- | --- | --- | --- | --- |
| **Zhejiang** | 1,913 | 90.75% | 0.00% | 9.25% |
| **Hunan** | 961 | 88.03% | 0.00% | 11.97% |
| **Guangdong** | 7,396 | 93.52% | 0.08% | 6.40% |

**Table S3: AMR Dataset: Different Expressions of “Wuhan Huanan Seafood Wholesale Market”**

| **Sample Raw Data for "Wuhan Huanan Seafood Wholesale Market" in AMR Dataset** | |
| --- | --- |
| **Location Name (Chinese)** | **Location Name (English Translation)** |
| 地址：武汉市江汉区华南海鲜市场东区二街35号 | Address: Wuhan Hanjiang Huanan Seafood Market East District 2nd Street No. 35 |
| 湖北省武汉市江汉区华南海鲜市场东区五街246号 | Hubei Wuhan Hanjiang Huanan Seafood Market East District 5th Street No. 246 |
| 华南海鲜市场 | Huanan Seafood Market |
| 华南海鲜市场周大周蟹行 | Huanan Seafood Market Zhoudazhou Crab Store |
| 武汉华南海鲜批发市场高氏水产批发部 | Wuhan Huanan Seafood Wholesale Market Gaoshi Aquatic Wholesale Department |
| 武汉市江汉区华南海鲜市场西十街525号 | Wuhan Hanjiang Huanan Seafood Market West 10th Street No. 225 |

**Table S4: False Positives in Clustering – Example One**

| **Raw Data** | **Processed Data** | | | |
| --- | --- | --- | --- | --- |
| **Market Name in AMR Dataset** | **Geographical (Prefecture)** | **Geographical (County)** | **Proper Name** | **Market Keyword** |
| 深圳市南山区华侨城东部市场 | 深圳市 | 南山区 | 华侨城东部 | 市场 |
| 深圳市南山区华侨城西部市场 | 深圳市 | 南山区 | 华侨城西部 | 市场 |

**Table S5: False Positives in Clustering – Example Two**

| **Raw Data** | **Processed Data** | | | |
| --- | --- | --- | --- | --- |
| **Market Name in AMR Dataset** | **Geographical (Prefecture)** | **Geographical (County)** | **Proper Name** | **Market Keyword** |
| 广东省佛山市三水区西南街百旺城市场 | 广东省佛山市 | 三水区 | 西南街百旺城 | 市场 |
| 广东省佛山市三水区西南街西南市场 | 广东省佛山市 | 三水区 | 西南街西南 | 市场 |
| 广东省佛山市三水区西南街德保市场 | 广东省佛山市 | 三水区 | 西南街德保 | 市场 |
| 广东省佛山市三水区西南街园林市场 | 广东省佛山市 | 三水区 | 西南街园林 | 市场 |

**Table S6: False Negatives in Clustering – Example One**

| **Raw Data** | **Processed Data** | | | |
| --- | --- | --- | --- | --- |
| **Market Name in AMR Dataset** | **Geographical (Prefecture)** | **Geographical (County)** | **Proper Name** | **Market Keyword** |
| 杭州市萧山区建设四路新农都水产市场 | 杭州市 | 萧山区 | 建设四路新农都 | 水产市场 |
| 杭州市萧山区新农都水产市场 | 杭州市 | 萧山区 | 新农都 | 水产市场 |
| 杭州萧山区新农都批发市场 | 杭州 | 萧山区 | 新农都 | 批发市场 |
| 杭州萧山区萧山新农都水产市场 | 杭州 | 萧山区 | 萧山新农都 | 水产市场 |
| 杭州市萧山区萧山新农都批发市场 | 杭州市 | 萧山区 | 萧山新农都 | 批发市场 |
| 杭州萧山区萧山新农都水产批发 | 杭州 | 萧山区 | 萧山新农都 | 水产批发 |
| 杭州市萧山区萧山新农都批发市场 | 杭州市 | 萧山区 | 萧山新农都 | 批发市场 |
| 杭州萧山区萧山新农都批发市场 | 杭州 | 萧山区 | 萧山新农都 | 批发市场 |
| 杭州萧山区萧山新农都物流中心强蓉水产行 | 杭州 | 萧山区 | 萧山新农都物流中心强蓉 | 水产行 |

**Table S7: False Negatives in Clustering – Example Two**

| **Raw Data** | **Processed Data** | | | |
| --- | --- | --- | --- | --- |
| **Market Name in AMR Dataset** | **Geographical (Prefecture)** | **Geographical (County)** | **Proper Name** | **Market Keyword** |
| 宁波路林市场 | 宁波 |  | 路林 | 市场 |
| 宁波路林水产批发 | 宁波 |  | 路林 | 水产批发 |
| 宁波市江北区路林市场 | 宁波市 | 江北区 | 路林 | 市场 |
| 宁波市路林市场 | 宁波市 |  | 路林 | 市场 |
| 浙江宁波江北路林市场 | 浙江宁波 | 江北 | 路林 | 市场 |
| 浙江省宁波市江北区路林市场 | 浙江省宁波市 | 江北区 | 路林 | 市场 |

**Table S8: Results of Clustering Accuracy Checks**

| **Clustering Accuracy Checks** | **Zhejiang** | **Cluster-level** | **Inaccurate Clusters** | **Total Clusters by Automation** | **Total Clusters by Manual Check** | **Accuracy Rate** |
| --- | --- | --- | --- | --- | --- | --- |
|  |  |  | 25 | 329 | 324 | 92.40% |
|  |  | **Record-level** | **Inaccurate Records** | **Total Records** | | **Accuracy Rate** |
|  |  |  | 210 | 1913 | | 89.02% |
|  | **Hunan** | **Cluster-level** | **Inaccurate Clusters** | **Total Clusters by Automation** | **Total Clusters by Manual Check** | **Accuracy Rate** |
|  |  |  | 9 | 176 | 172 | 94.90% |
|  |  | **Record-level** | **Inaccurate Records** | **Total Records** | | **Accuracy Rate** |
|  |  |  | 63 | 961 | | 93.44% |
|  | **Guangdong** | **Cluster-level** | **Inaccurate Clusters** | **Total Clusters by Automation** | **Total Clusters by Manual Check** | **Accuracy Rate** |
|  |  |  | 99 | 1198 | 1195 | 91.70% |
|  |  | **Record-level** | **Inaccurate Records** | **Total Records** | | **Accuracy Rate** |
|  |  |  | 594 | 7396 | | 91.97% |

**Table S9: Failure Rates by Data Source Type for AMR Dataset Tests in Beijing**

| **Failure Rate - State AMR** | **Failure Rate - Provincial AMR Organizations** | **Failure Rate - Prefecture AMR Organizations** |
| --- | --- | --- |
| 0.59% | 1.24% | 1.05% |
